# Cdc42 Regulates the Expression of Cytoskeleton and Microtubule Network Proteins to Promote Invasion and Metastasis of Progeny Cells Derived from CoCl_2_-induced Polyploid Giant Cancer Cells

**DOI:** 10.7150/jca.85032

**Published:** 2023-06-26

**Authors:** Minying Zheng, Lankai Chen, Junjie Fu, Xiaohui Yang, Shuo Chen, Wenzheng Fu, Yuwei Li, Shiwu Zhang

**Affiliations:** 1 Department of Pathology, Tianjin Union Medical Center, Nankai University, Tianjin, 300121, P.R. China.; 2 Nankai University School of Medicine, Nankai University, Tianjin, 300071, P.R. China.; 3 Department of Colorectal Surgery, Tianjin Union Medical Center, Tianjin, 300121, P.R. China.; 4 State Key Laboratory of Medicinal Chemical Biology, NanKai University, Tianjin, 300071, P.R. China.

**Keywords:** PGCCs, Cdc42, stathmin, PAK1, cathepsin B, cathepsin D

## Abstract

**Purpose:** Our previous studies have shown that CoCl_2_ can induce the formation of polyploid giant cancer cells (PGCCs) and PGCCs could produce progeny cells via asymmetric division. In this study, the molecular mechanism by which PGCCs generate progeny cells with high invasion and migration abilities was explored.

**Methods:** In this study, PGCCs induced by CoCl_2_ produced progeny cells via asymmetric division, which was observed dynamically using laser scanning confocal microscopy. Cell cycle in LoVo and Hct116 before and after CoCl_2_ treatment was analyzed by flow cytometry. Cell function experiments, co-immunoprecipitation, mass spectrometry analysis, ML141 treatment, western blotting, and siRNA transfection experiments were used to demonstrate that Cdc42/PAK1 was involved in the regulation of cytoskeleton expression. The proliferation, migration, and invasion abilities of PGCCs and progeny cells were compared in PGCCs and progeny cells with and without inhibiting the expression of Cdc42 and PAK1.

**Results:** G2/M phase arrest appeared in CoCl_2-_treated LoVo and Hct116 cells. After CoCl_2_ treatment, an increased expression of Cdc42 and PAK1 led to a decrease in the expression of stathmin and an increase in the expression of phosphorylated stathmin, which is located in the nucleus of PGCCs and progeny cells. PTPN14 negatively regulates the expression of PAK1 and p38MAPK. Low levels of PTPN14 expression, a downstream regulatory protein of stathmin, endows progeny tumor cells generated by PGCCs with the ability to invade and metastasize. The expression of PKA1α, cathepsin B, and D increased in CoCl_2_-treated cells compared with that in the control cells, associated with the infiltration and migration of PGCCs with their progeny cells.

**Conclusion:** CoCl_2_-induced overexpression of Cdc42 plays a critical role in increasing the infiltration and migration abilities of PGCCs and progeny cells by regulating cytoskeleton protein expression.

## Introduction

According to the latest global cancer data released by the World Health Organization's International Agency for Research on Cancer (IARC) in 2021, colorectal cancer (CRC) is the third most common cancer worldwide. The recurrence and metastasis of CRC are major causes of poor prognosis in patients, which is associated with high rates of morbidity and mortality 1, 2.

Polyploid giant cancer cells (PGCCs) have been observed for over a century 3, 4. It was reported that PGCCs can be induced by cobalt chloride (CoCl_2_), a type of hypoxia mimic, *in vitro*. PGCCs are a special subpopulation of cancer cells, which closely associate with the heterogeneity of solid tumors 5, 6. PGCCs not only express markers of cancer stem cells (CSCs) but also have characteristics of CSCs 5. PGCCs are a key factor contributing to the heterogeneity and abnormal chromosomal structure of solid human cancers. The number of PGCCs in tumor cells is closely related to pathological grade, clinical stage, chemotherapy resistance, patient prognosis, and recurrence 7. PGCC formation can be induced by many endogenous and exogenous stimuli, including chemical reagents, radiation, hypoxia, arsenic trioxide, triptolide, and viral infection 8-12. PGCCs produce progeny cells via asymmetric division. Progeny cells highly express epithelial-mesenchymal transition-related proteins and have a stronger ability to invade and metastasize 13, 14. Many tumor invasion- and metastasis-related proteins are highly expressed in PGCCs and their progeny cells. However, the molecular mechanisms involved in the high invasion and metastatic ability of progeny cells derived from PGCCs remain unclear.

In our previous studies, we showed that cell division cycle 42 (Cdc42) and cell cycle-related proteins are associated with the generation of progeny cells from PGCCs by regulating cytoskeletal changes and mitotic processes during cell division 5. Based on iTRAQ-based proteomic analysis, we showed that Cdc42 is significantly upregulated in PGCCs and progeny cells 15. Cdc42 belongs to the Rho GTPase family and plays an important role in malignant tumors by regulating the cytoskeleton and microtubule dynamics, cell polarity, and cell cycle progression. The optimal levels of activation of Cdc42, Rho and Rac were required in cellular invasion 16. Cdc42-controlled filopodia may be involved in extra-cellular matrix recognition. In addition, the activation of Cdc42 can induce the formation of smaller adhesion complexes localized to the cell periphery, which is likely to be critical for the complex process of cell invasion 17, 18. PAK1 belongs to the PAKs family and is involved in cytoskeleton remodeling, cell motility, proliferation, apoptosis, and mitotic abnormalities 19. PAK1 is not only an effector protein of Cdc42 but also a scaffold protein that can activate Cdc42. The mutual activation of PAK1 and Cdc42 contributes to the directed migration of F-actin 20. In normal human cells, PAK1 exists in a dimeric form and in an inhibitory state. When the GTPase binds to Cdc42 or RAC1, PAK1 dissociates into a monomeric form and is activated 21. PTPN14 is a downstream regulatory protein of stathmin (STMN1) and can negatively regulate the expression of PAK1 and mitogen-activated protein kinase (p38MAPK).

In this study, we showed that increased Cdc42 and PAK1 expression can decrease the expression of STMN1 and increase the expression of phosphorylated stathmin, which is located in the nucleus of PGCCs and progeny cells to regulate cytoskeletal remodeling. The low expression of PTPN14 in cells after CoCl_2_ treatment was associated with the infiltration and migration of progeny cells by regulating the expression of PAK1.

## Materials and Methods

### Cell culture

Both LoVo and Hct116 colorectal cancer cells were obtained from the American Type Culture Collection (USA). The cells were cultured in 1640 (Gibco, Thermo Fisher Scientific, Suzhou, China) medium containing 10% fetal bovine serum (FBS) (Gibco, Life Technologies, New Zealand) and 1% penicillin and streptomycin and incubated at 37 ℃ and 5% carbon dioxide.

### Formation of PGCCs

LoVo and Hct116 cell lines were cultured in T25 cell culture flasks until the confluency reached 80-90%. CoCl_2_ (450 μM) was added to the two cell lines for 48-72 h, respectively. Most of the diploid tumor cells in the culture flask died, and only a few large tumor cells remained. The flask was rinsed with phosphate-buffered saline, and the cells were incubated in 1640 medium without CoCl_2_. Between 10 and 14 days after the first treatment with CoCl_2_, PGCCs began to produce progeny cells via asymmetric division.

### Western blotting

The collected cell pellets including the control and CoCl_2_-treated cells, were harvested and lysed with an appropriate amount of RIPA lysis buffer (phenylmethanesulfonyl fluoride was added to the lysis buffer, 1:100) on ice for 30 min, vortexed, and then centrifuged at 14,000 rpm for 20 min. After the centrifugation, the supernatant was collected, an appropriate amount of 5× loading buffer was added, and the mixture was boiled at 100 ℃ for 10 min. According to the molecular weight of the protein, the corresponding concentration of the separation gel (8%, 10%, and 12%) was prepared for electrophoresis and transferred to a polyvinylidene difluoride (PVDF) membrane. Next, the PVDF membrane was placed in 5% milk prepared in Tris-HCL and tween buffer salt solution and blocked for 1-2 h at room temperature. According to the molecular weight of proteins, PVDF membrane was cut prior to hybridisation with different antibodies. The corresponding first antibodies were added and incubated overnight at 4 °C. The next day, PVDF membranes were incubated with the corresponding secondary antibodies for 1-2 h at room temperature. Detection was performed using the ChemiDoc imaging system (BioRad, USA), and the experimental data were analyzed by Image J. All western blotting analyses were conducted independently in triplicate.

### Immunocytochemical (ICC) staining

LoVo and Hct116 control and CoCl_2_-treated cells, were allowed to grow for 24-48 h in a six-well plate. When a cell density of 70% was reached, the cells were fixed with ice-cold methanol for 30 min. After fixation, the cells were incubated with a peroxidase inhibitor for 15 min. The non-specific protein-binding background was blocked with anti-goat serum for 20 min. Then, the slides were incubated with the corresponding primary antibodies overnight at 4 °C. The next day, 1-2 drops of biotin-labeled goat anti-mouse/rabbit IgG polymer were added, and the slides were incubated at room temperature for 30 min. The diamnobenzidene color developing solution was used to observe the brown particles under the microscope, and the nuclei were stained with hematoxylin for 30 s. Finally, the slides were dehydrated using an alcohol gradient, cleared with xylene, and mounted with neutral gum.

### Co-immunoprecipitation (Co-IP) assay and mass spectrometry

The cells were collected with a cell scraper once a confluency of 80-90% was reached. IP-specific lysis buffer containing 1 mM PMSF, according to the amount of cell pellet, was added, and the control and CoCl_2_-treated cells were lysed on ice for 30 min. Then, 30 μL of A/G agarose homogenate of agar glycoprotein beads and 500 μL of IP-specific lysis buffer were added to pre-cooled EP tubes. After lysis, the tubes were centrifuged at 14,000 rpm for 20 min at 4 °C, and the supernatant was transferred to an EP tube containing A/G agarose homogenate of agar glycoprotein beads and shaken at 4 °C for 30 min to remove background. After incubation, the supernatant was divided into three parts: one part was used to detect the total protein level (“input”), and primary antibodies of rabbit IgG and target protein were added the other two tubes, respectively, and kept at 4 °C overnight. On the following day, the A/G agarose homogenates of agar glycoprotein beads were washed. Those samples containing rabbit IgG and primary antibodies of target protein were transferred to the newly washed column and incubated at 4 °C for 2 h. After incubation, the supernatant was discarded, and 500 µL of IP-specific lysis buffer was added to wash the beads five times. Lastly, western blotting was performed to analyze the samples.

### Transient siRNA transfection

Three siRNA interfering sequences of Cdc42 and PAK1 (Supplementary table 1), including one positive interfering sequence, one nonsense sequence, and one fluorescent interfering sequence, were ordered from GenePharma (Shanghai, China). Transfection was performed when the density of the LoVo and Hct116 control cells and CoCl_2_-treated cells in the six-well plate reached 40-60%. Next, 50 μL of Opti-MEM (Gibco, USA), 50 μL of interfering sequence, and 5μL of Lipofectamine 2000 (Invitrogen, Carlsbad, CA, USA) reagent were added to each well of a six-well plate, followed by incubation for 6-8 h. Cell proteins were collected for subsequent western blotting validation.

### Transient transfection of PTPN14 overexpression plasmid

The overexpressed exogenous gene *PTPN14* was purchased from GENEWIZ. The specific transfection steps were as follows: PGCCs with progeny cells of LoVo and Hct116 were cultured in six-well plates and used for overexpression transient transfection once the cells reached a confluency of 40-60%. The cells were cultured in RPMI 1640 medium without antibiotics. The transfection mixture (110 μL), containing 10 μL of Lipofectamine 2000 transfection reagent, 90 μL of Opti-MEM medium, and 10 μL of PTPN14 overexpression transfection plasmid, was added to the wells for 7 h. After incubation, the medium in the wells was discarded and replaced with normal medium for 48 h, and the transfected cells were collected for western blot analysis.

### Immunofluorescence (IF) staining

F-actin was tested using direct IF staining. Cells at a density of 80-90% were digested and plated in the six-well plates containing cover slides. The cells cultured on the cover slides reached a density of 50% and were fixed with 4% paraformaldehyde for 15 min. Phalloidin was added to the medium at 37°C for 30 min in the dark. DAPI was used to stain the nuclei, after which the cells were photographed using a fluorescence microscope. For Cdc42 and tubulin IF staining, the cells in the six-well plate grew to a density of 50% and were then fixed with 4% paraformaldehyde for 15 min. After fixation, cells were treated with 0.5% TritonX-100 for 15 min and blocked with 5% BSA for 60 min. Cdc42 (1:100; Abcam, USA) and anti-tubulin (1:100; CST, USA) antibodies were added and incubated overnight at 4 °C. On the following day, a fluorescent-labeled secondary antibody (1:1000; anti-rabbit IgG Fab2 Alexa Flour488) was added. The nuclei were stained with DAPI for 15 min, and the cells were photographed.

### Application of small molecule inhibitor ML141

ML141 (20 μM) was added to the medium of the LoVo and Hct116 control and the CoCl_2_-treated cells. After 24 h of treatment, the cell pellets were collected and lysed, and the proteins were collected for subsequent western blotting analysis.

### Transwell invasion and migration assay

For invasion experiments, 600 μL of medium containing 20% serum was added to a 24-well plate, and a Transwell gel-coating chamber was placed in the well. Next, 200 μL of serum-free cell suspension containing 5 × 10^5^ LoVo and Hct116 control and the CoCl_2_-treated cells was added to the cell incubator and incubated for 24 h. The cells were fixed with anhydrous methanol and stained with 0.1% crystal violet. The membrane at the bottom of the chamber was cut off using a scalpel, photographed, and counted under a microscope. For the migration assay, the cells were cultured in a chamber without gel coating.

### Plate colony formation assay

The control and CoCl_2_-treated LoVo and Hct116 cells were counted using a cell counter. A plate colony formation assay was performed in a 12-well plate. One milliliter of medium containing serum was added to the well. The cells (30, 60, and 120) were cultured in the wells (three replicate wells for each group of cells). The 12-well plate was placed in an incubator for two weeks at 37 °C. The white cell mass was visible, fixed with anhydrous methanol, and stained with 0.1% crystal violet. Subsequently, the number of cells was counted under a microscope.

### Real-time quantitative PCR (RT-qPCR)

Total RNA from LoVo and Hct116 cells before and after CoCl_2_ treatment was extracted using the TRIzol method, and cDNA was synthesized using a reverse transcription kit (Shanghai Yisheng). cDNA was amplified and detected using the Hieff UNICON Universal Blue qPCR SYBR Green MasterMix. The PCR primer sequences were presented in supplementary table 2. This experiment used 20-μL reaction systems. The RT-qPCR results were analyzed by calculating 2^-ΔΔCT^.

### Laser scanning confocal microscope (LCM)

F-actin was stained using a SiR-actin Kit (CY-SC001) (Spirochrome, USA). First, the stock solution was prepared at a concentration of 100 nM. Then, 10 μM efflux pump inhibitor was added and incubated for 12 h. Nuclear Green^TM^ LCS1 was formulated at a concentration of 3 μM, added to the cells, and incubated for 45 min. After incubation, the cells were periodically photographed using a confocal microscope.

### Flow cytometry analysis of cell cycle

The CoCl_2-_treated and control LoVo and Hct116 cells were collected and fixed with 70% ethanol at -20°C overnight. Cells were stained with 0.5 ml of PI/RNase staining buffer (BD Biosciences, USA) for 15 minutes at room temperature and analyzed by flow cytometry (BD FACSCalibur™, BD Biosciences).

### Statistical analysis

Each experiment was repeated at least three times. Statistical significance was assessed by comparing mean values (6 SD) using the Student *t* test for independent groups as follow: *, *P* < 0.05; **, *P* < 0.01; ***,* P* < 0.001 and not significant (NS).

## Results

### Formation of PGCCs with progeny cells induced by CoCl_2_

**Figure 1:**
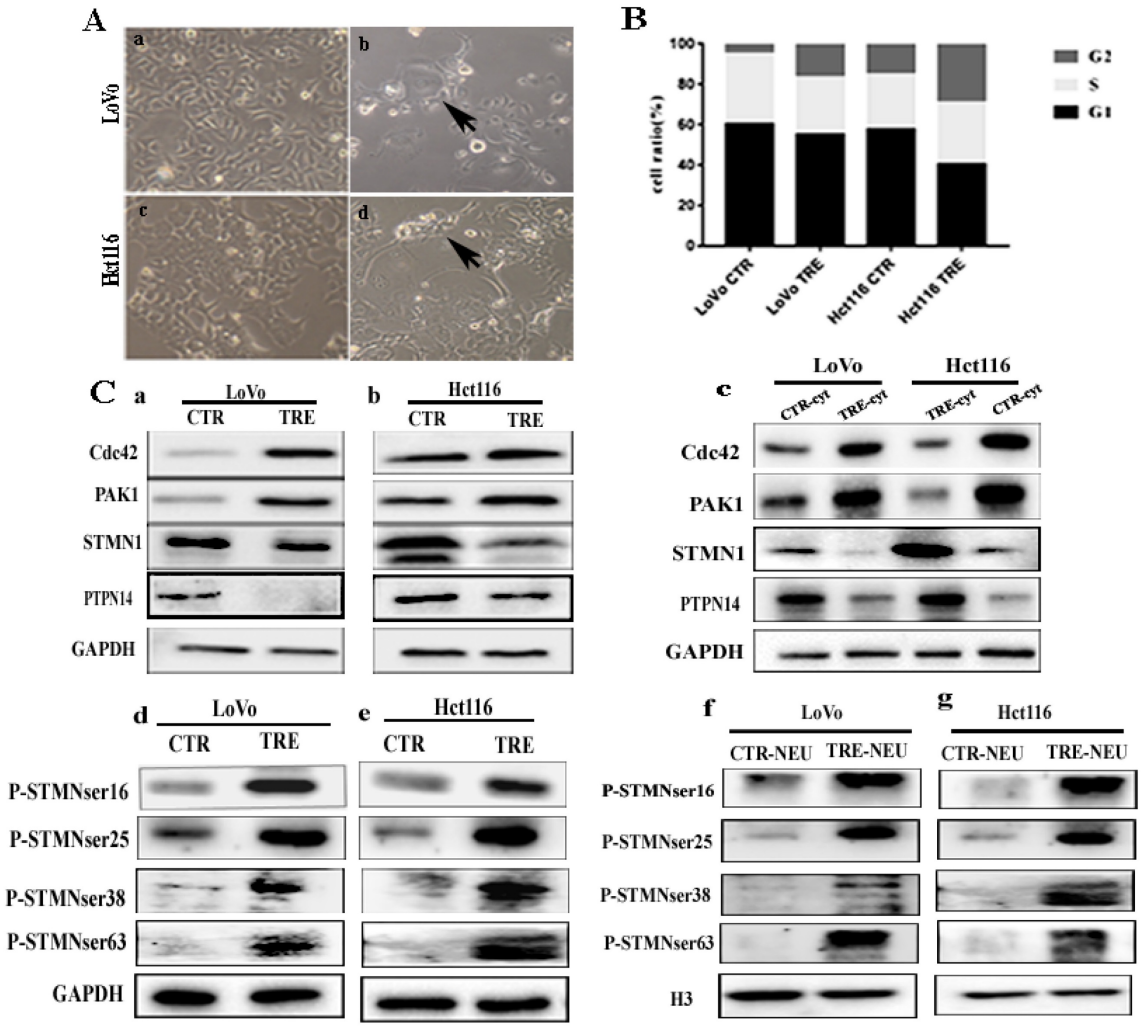
** A.** Formation of PGCCs after CoCl_2_ treatment in LoVo and Hct116 colorectal cancer cell lines (200×). (a) LoVo control cells, (b) CoCl_2_-treated LoVo cells (black arrow indicates PGCCs), (c) Hct116 control cells, and (d) CoCl_2_-treated Hct116 cells, black arrow points to PGCCs.** B.** Columnar percentage plot showing the ratio of cells at G1, S, and G2 stages of cell cycle in LoVo and Hct116 cells before and after CoCl_2_ treatment. **C.** (a,b) Western blotting result of Cdc42, PAK1, STMN1, and PTPN14 in LoVo and Hct116 control and CoCl_2_-treated cells. (c) Cytoplasmic expression of Cdc42, PAK1, STMN1, and PTPN14 in LoVo and Hct116 control and CoCl_2_-treated cells. (d, e) Expression of four phosphorylation sites of STMN1 in LoVo and Hct116 control and CoCl_2_-treated cells. (f, g) Nuclear expression of the four phosphorylation sites of STMN1 in LoVo and Hct116 control and CoCl_2_-treated cells.

The LoVo and Hct116 cells were treated with CoCl_2_ concentration of 450 μmol/L for 48-72 h. After CoCl_2_ treatment, most small-sized cells were dead, and the remaining tumor cells (PGCCs) were 3-5 times larger than the small-sized cells (Fig. 1A). Results of cell cycle analysis showed that more cells were blocked in G2/M phase in CoCl_2-_treated LoVo and Hct116 cells compared with the control cells. The proportion of PGCCs was approximately 16.68% and 29.10% in CoCl_2-_treated LoVo and Hct116 cells, respectively (Fig. 1B and Fig. S1A).

### Expression levels of Cdc42, PAK1, PTPN14, STMN1, and phosphorylated STMN1 and their four phosphorylation sites

**Figure 2:**
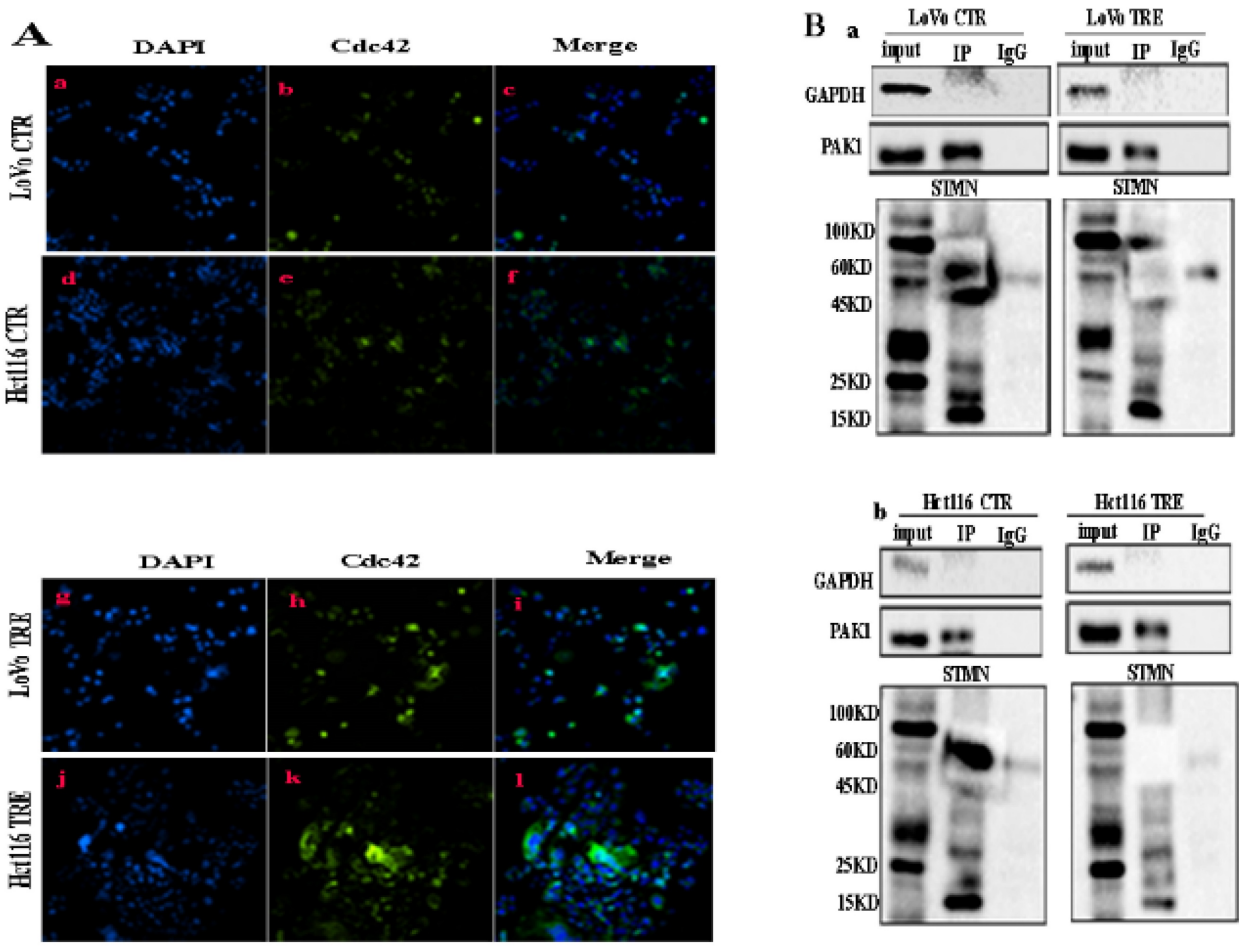
**A** (a-f) IF staining of Cdc42 in LoVo and Hct116 control cells (200×) and (g-l) IF staining of Cdc42 in CoCl_2_-treated LoVo and Hct116 cells (200×).** B.** The interaction between PAK1 and STMN1 was verified by Co-IP in LoVo (a) and Hct116 (b) control and CoCl_2_-treated cells. CTR: control cells; TRE: CoCl_2_-treated cells; P-STMN1: phosphorylated stathmin

In CoCl_2_-treated cells, the expression levels of Cdc42 and PAK1 were significantly higher than those in the control cells (Fig. 1C a, b). Cytoplasmic Cdc42 and PAK1 were upregulated in CoCl_2_-treated cells compared with the control cells (Fig. 1C c). Results of ICC staining showed that Cdc42 and PAK1 were located in the cytoplasm, and the staining degree of CoCl_2_-treated cells was significantly higher than that of the control cells (Fig. S1B a-h). In addition, the western blotting results showed that the expression level of STMN1 in CoCl_2_-treated was significantly lower than that in control cells (Fig. 1C a, b). Cytoplasmic STMN1 were downregulated in CoCl_2_-treated cells compared with the control cells (Fig. 1C c). ICC staining revealed that STMN1 was expressed in the cytoplasm (Fig. S1B i-l). P-STMNser16 was mainly expressed in the nucleus of PGCCs and progeny cells, and the staining degree was significantly higher than that in the control cells (Fig. S1C m-p). However, the expression levels of phosphorylated STMN1 at four phosphorylation sites (Ser16, Ser25, Ser38, and Ser63) were elevated in CoCl_2_-treated cells (Fig. 1C d, e). By examining nuclear proteins, the western blotting results showed that the four phosphorylation sites of STMN1 (Ser16, Ser25, Ser38, and Ser63) appeared in the nucleus, and the expression levels in CoCl_2_-treated cells were significantly higher than those in control cells (Fig. 1C f, g). The western blotting results showed that the expression of PTPN14 was downregulated in CoCl_2_-treated cells compared to that in the control cells (Fig. 1C a, b). The cytoplasmic expressions of PTPN14 was decreased in cells after CoCl_2_ treatment compared with the control cells (Fig. 1C c). ICC staining verified that PTPN14 was localized in the cytoplasm, and the expression of PTPN14 in control cells was higher than that in CoCl_2_-treated cells (Fig. S1B q-t). Quantitative analysis showed that the expression of Cdc42, PAK1, STMN1, PTPN14 and phosphorylated STMN1 in CoCl_2_-treated cells was significantly different from that in the control cells, consistent with the western blotting results (Fig. S2A). In addition, IF staining showed that Cdc42 expression was localized in the cytoplasm, and the staining degree in CoCl_2_-treated cells was significantly higher than that in the control cells (Fig. 2A).

### Validation of the interaction between Cdc42, PAK1, and STMN1 by Co-IP and combined mass spectrometry

**Figure 3:**
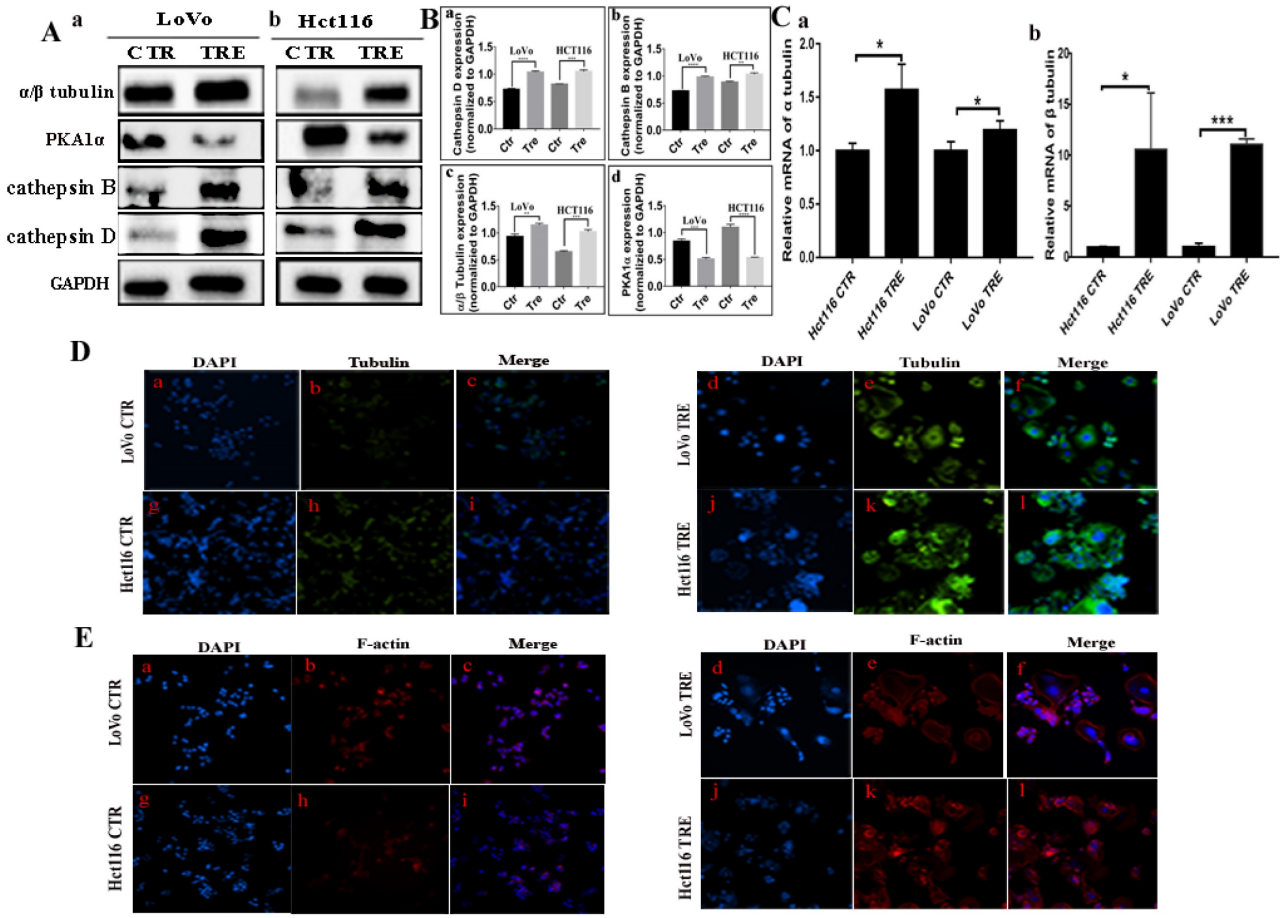
** A.** Western blotting results for α/β tubulin, PKA1α, cathepsin B, and cathepsin D in LoVo (a) and Hct116 (b) control and CoCl_2_-treated cells. **B.** Histograms show the total protein expression of cathepsin B (a), cathepsin D (b), α/β tubulin (c), and PKA1α (d) in LoVo and Hct116 control and CoCl_2_-treated cells. **C.** The mRNA levels of tubulin were examined by RT-PCR in LoVo and Hct116 control and CoCl_2_-treated cells. (a) The mRNA levels of α tubulin. (b) The mRNA levels of β tubulin. **D.** IF staining of α/β tubulin of LoVo and Hct116 control and CoCl_2_-treated cells (200×). (a-c) LoVo control cells, (d-f) CoCl_2_-treated LoVo cells, (g-i) Hct116 control cells, (j-l) and CoCl_2_-treated Hct116 cells. **E.** IF staining of F-actin in LoVo and Hct116 control and CoCl_2_-treated cells (200×). (a-c) LoVo control cells, (d-f) CoCl_2_-treated LoVo cells, (g-i) Hct116 control cells, and (j-l) CoCl_2_-treated Hct116 cells. CTR: control cells; TRE: CoCl_2_-treated cells.

Co-IP was performed using PAK1 as a unk bait protein. As shown in Fig. 2B, GAPDH bands appeared in the input group, while no bands appeared in the IP and IgG groups, indicating that the immunoprecipitation samples in the experimental group and the negative control group did not have non-specific heteroband effects. Protein bands for PAK1 appeared in both the input and IP groups, indicating that the results were credible (Fig. 2B a, b). STMN1 bands appeared in the input and IP groups of the two cell lines, and the expression of STMN1 in PGCCs with progeny cells was lower than that in the control cells (Fig. 2B a, b). Immunoprecipitation combined with mass spectrometry analysis showed that Cdc42 interacts with cathepsin B, cathepsin D, α/β tubulin, and PKA1α (Fig. S2B, Table S3 and S4). The results of the western blotting showed that the expression of cathepsin B, cathepsin D, and α/β tubulin were higher in PGCCs with progeny cells than that in the control. The expression of PKA1α in PGCCs with progeny cells was lower than that in the control group (Fig. 3A a, b). The quantitative analysis of the western blotting grave values revealed the expression of cathepsin B, cathepsin D, α/β tubulin, and PKA1α in the control cells and PGCCs with progeny cells of LoVo and Hct116 (Fig. 3B a-d). In addition, quantitative real-time PCR revealed that the mRNA level of α/β tubulin was higher and the mRNA level of PKA1α was lower in CoCl_2_-treated cells compared with the control cells (Fig. 3C and Fig. S2C). To further verify the subcellular localization of cathepsin B, cathepsin D, α/β tubulin, and PKA1α, ICC staining was performed on the cells before and after CoCl_2_ treatment. As shown in figure S1, α/β tubulin (Fig. S1C a-d), cathepsin B (Fig. S1C e-h), PKA1α (Fig. S1C i-l), and cathepsin D (Fig. S1C m-p) were located in the cytoplasm, and the expression of α/β tubulin, cathepsin B, and cathepsin D in the cytoplasm of PGCCs with progeny cells was higher than that that in control cells (Fig. S1C). The expression of PKA1α in PGCCs with progeny cells was slightly lower than that in control group cells (Fig. S1C). We further verified the subcellular localization and quantification of α/β tubulin expression using IF staining. As shown in Fig. 3D, α/β tubulin was completely localized in the cytoplasm of PGCCs with progeny cells and control cells, and the fluorescence intensity in PGCCs with progeny cells was significantly higher than that in the control cells. As Cdc42 can regulate the expression of cytoskeleton-related proteins, F-actin IF staining in cells before and after CoCl_2_ treatment was performed, the results of which showed that PGCCs with progeny cells showed strong fluorescence intensity (Fig. 3E).

### Knockdown of Cdc42 expression regulates the expression of PAK1, STMN1, PTPN14, and Cdc42-related proteins

**Figure 4:**
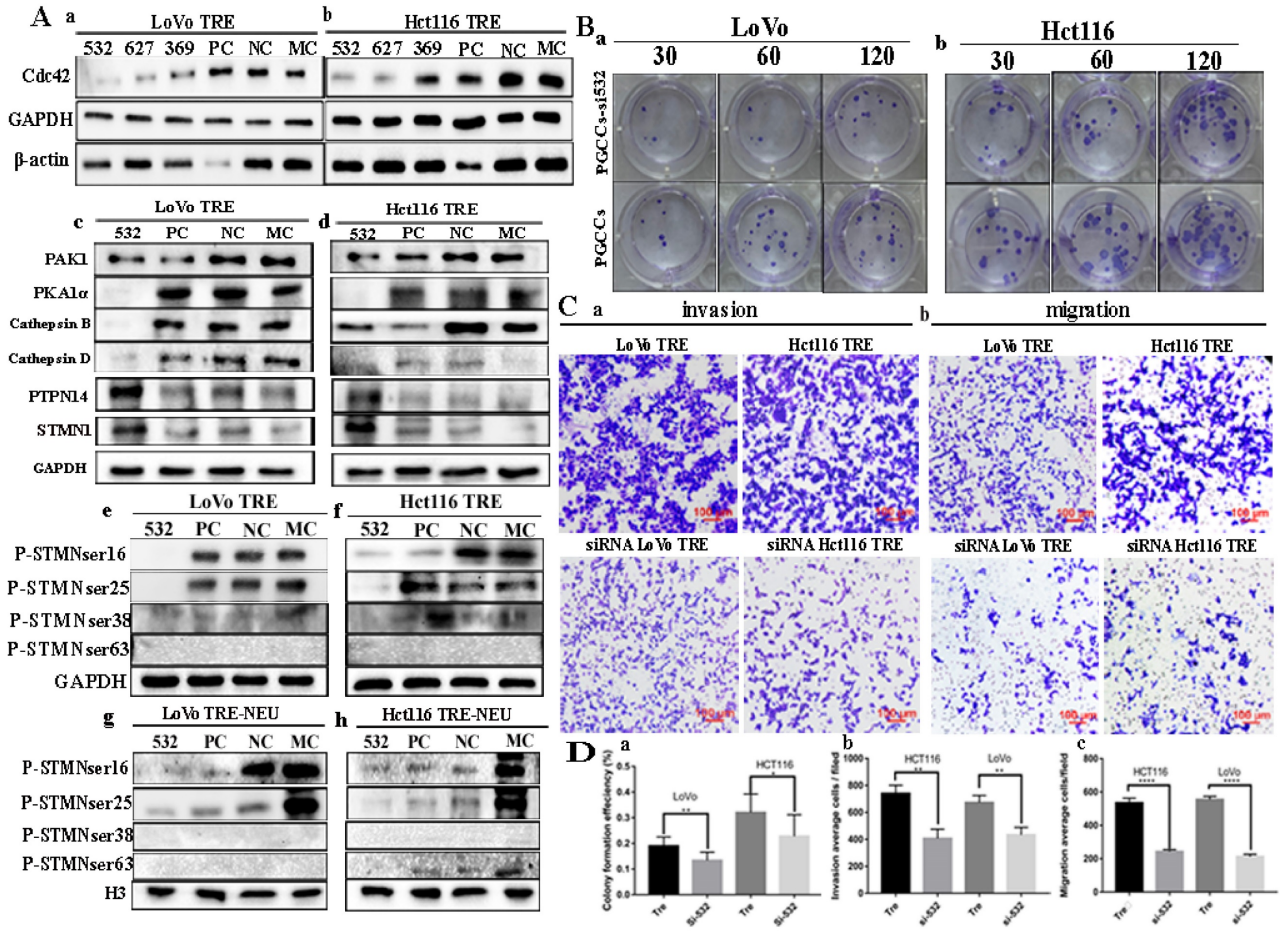
** A.** Western blotting results in cells after Cdc42 knockdown. (a, b) Expression of Cdc42 and β-actin in CoCl_2_-treated LoVo and Hct116 cells transfected with siRNA Cdc42-532, 627, 369, siRNA control, and negative control, respectively. (c, d) Total protein expression of Cdc42, PAK1, PKA1α, cathepsin B, cathepsin D, PTPN14, and STMN1 after transfection of siRNA Cdc42-532, PC, NC, and MC in LoVo and Hct116 CoCl_2_-treated cells. (e, f) Expression of four phosphorylation sites of STMN1 in LoVo and Hct116 CoCl_2_-treated cells transfected with siRNA Cdc42-532, siRNA control, and negative control, respectively. (g, h) Nuclear expression of four phosphorylation sites of STMN1 in LoVo and Hct116 CoCl_2_-treated cells transfected with siRNA Cdc42-532, PC, NC, and MC, respectively.** B.** Colony formation experiments of CoCl_2_-treated LoVo (a) and Hct116 (b) before and after Cdc42 knockdown. **C.** Transwell experiment of CoCl_2_-treated LoVo and Hct116 cells before and after Cdc42 knockdown (40×). (a) Cell invasion assay and (b) cell migration assay. **D.** (a) Clone formation efficiency of CoCl_2_-treated LoVo and Hct116 cells before and after Cdc42 knockdown. (b) Statistical result of the average invasive cell number of CoCl_2_-treated LoVo and Hct116 cells before and after Cdc42 knockdown. (c) Statistical result of the average migration cell number of CoCl_2_-treated LoVo and Hct116 cells before and after Cdc42 knockdown. CTR, control cells; TRE, CoCl_2_-treated cells; PC, positive control; NC, negative control; MC, mock control.

The expression of Cdc42 was inhibited by siRNA transfection, and the expression levels of Cdc42-related proteins, including PAK1, STMN1, PTPN14, PKA1α, cathepsin B, cathepsin D, were detected by western blotting. Three transfection sequences (532, 627, and 369) were used, and the western blotting results showed that the siRNA Cdc42-532 sequence had the strongest inhibitory efficiency in LoVo and Hct116 PGCCs with progeny cells (Fig. 4A a, b). The expression of PAK1, cathepsin B and cathepsin D was decreased in PGCCs with progeny cells, while the expression of STMN1, PKA1α, and PTPN14 was increased (Fig. 4A c, d) in PGCCs with progeny cells after Cdc42 knockdown. However, the phosphorylation of STMN1 decreased after reducing the expression of Cdc42. Different phosphorylation sites of STMN1 showed different expression levels. The expression of Ser16 and Ser25 sites of STMN1 decreased, whereas the phosphorylation levels of Ser38 and Ser63 were not expressed. After the extraction of the nuclear protein, the nuclear expression of Ser16 and Ser25 of STMN1 decreased, while the phosphorylation levels of Ser38 and Ser63 were not expressed in the nucleus after Cdc42 was knocked down (Fig. 4A e-h). In addition, we examined the proliferation, migration, and invasion abilities of PGCCs with progeny cells before and after Cdc42 knockdown. The results of plate cloning experiments showed that the proliferation ability of PGCCs and progeny cells decreased after Cdc42 knockdown (Fig. 4B a, b). Transwell experiments confirmed that PGCCs with progeny cells had reduced migratory and invasive abilities after Cdc42 knockdown (Fig. 4C a, b). The quantitative analysis of the changes in the migration, invasion, and proliferation abilities showed that there were significant differences between PGCCs and their progeny cells before and after Cdc42 knockdown (Fig. 4D a-c).

### Expression of Cdc42-related proteins and their effects on the proliferation, metastasis, and invasion abilities after ML141 treatment

**Figure 5:**
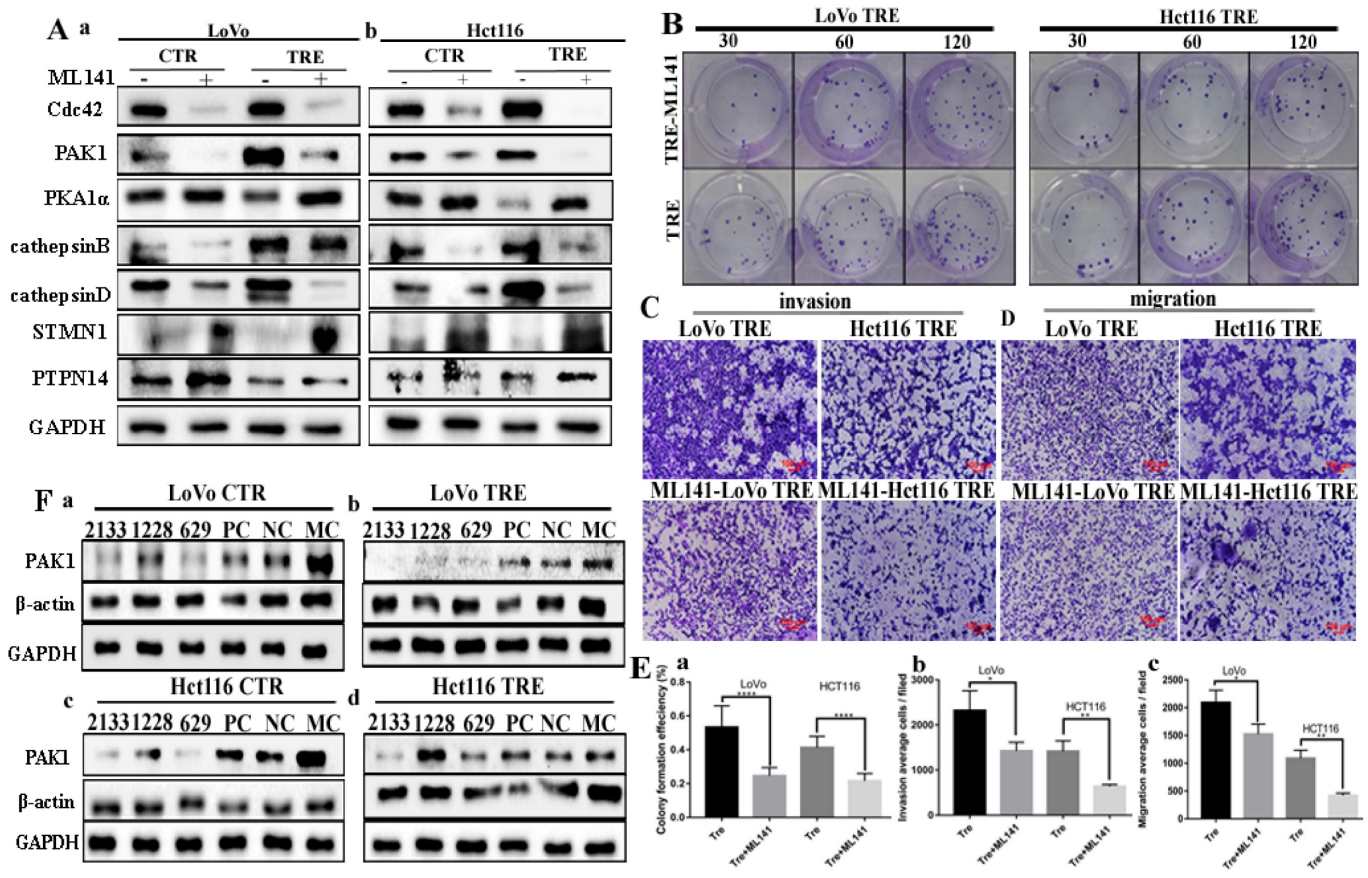
** A.** Total protein expression of Cdc42, PAK1, PKA1α, cathepsin B, cathepsin D, PTPN14, and STMN1 in LoVo (a) and Hct116 (b) control and CoCl_2_-treated cells before and after ML141 treatment. **B.** Clonal formation of CoCl_2_-treated LoVo and Hct116 before and after ML141 treatment. **C.** Cell invasion assay of CoCl_2_-treated LoVo and Hct116 cells before and after ML141 treatment (40×).** D.** Cell migration experiments of LoVo and Hct116 CoCl_2_-treated cells before and after ML141 treatment (40×). **E.** Histograms of the mean cell numbers of CoCl_2_-treated LoVo and Hct116 cells in clonogenic efficiency (a), invasion (b), and migration (c) experiments before and after ML141 treatment.** F.** Expression of PAK1 after transfection with siRNA PAK1-2133, 1228, 629, PC, NC, and MC in LoVo control (a), CoCl_2_-treated LoVo cells (b), Hct116 cells (c), and CoCl_2_-treated Hct116 cells (d). CTR, control cells; TRE, CoCl_2_-treated cells; PC, positive control; NC, negative control; MC, mock control.

The expression levels of Cdc42-related proteins and changes in the proliferation, migration, and invasion abilities were detected using the small molecule inhibitor ML141. ML141 cannot non-competitively inhibit the activity of Cdc42. In the control cells and PGCCs with progeny cells of LoVo and Hct116 cells treated with ML141 for 24 h, the western blotting results showed that Cdc42 was significantly inhibited. The expression levels of the Cdc42-related proteins PAK1, cathepsin B, and cathepsin D were downregulated, and the expression of PKA1α, PTPN14, and STMN1 increased after ML141 treatment (Fig. 5A a, b). The quantitative analysis of the western blotting gray values is presented in supplementary figure 2D. In addition, we examined the proliferation, migration, and invasion abilities of LoVo and Hct116 PGCCs with progeny cells before and after ML141 treatment. The results of plate colony formation experiments showed that the proliferation ability of PGCCs with progeny cells decreased after ML141 treatment (Fig. 5B). Transwell experiments showed that the invasion and migration abilities of PGCCs and their progeny cells decreased after ML141 treatment (Fig. 5C and 5D). Quantitative analysis showed that the proliferation, invasion, and migration abilities decreased in PGCCs with progeny cells after ML141 treatment compared to those without ML141 treatment, and the differences were statistically significant (Fig. 5E a-c).

### Expression of STMN1, phosphorylated STMN1, PTPN14, cathepsin B, cathepsin D, α/β tubulin, and p38MAPK before and after PAK1 knockdown

**Figure 6:**
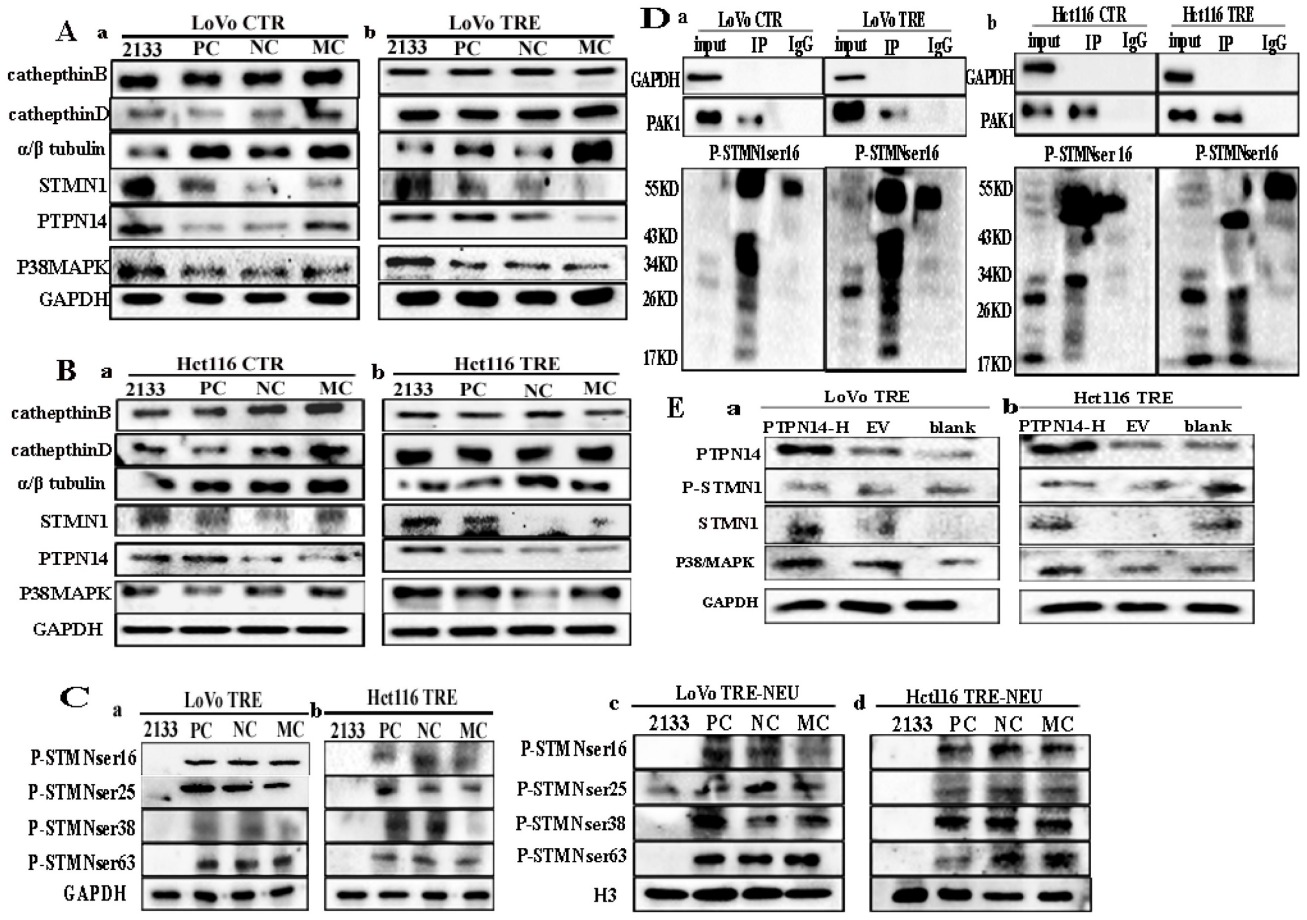
** A.** (a,b) Total protein expression of cathepsin B, cathepsin D, α/β tubulin, PTPN14, STMN1, and P38MAPK after the knockdown of PAK1 expression in LoVo control and CoCl_2_-treated cells. **B.** (a,b) Total protein expression of cathepsin B, cathepsin D, α/β tubulin, PTPN14, STMN1, and P38MAPK after the knockdown of PAK1 expression in Hct116 control and CoCl_2_-treated cells. **C.** (a, b) Expression of four phosphorylation sites of STMN1 in CoCl_2_-treated LoVo and Hct116 cells transfected with siRNA PAK1-2133, PC, NC, and MC, respectively. (c, d) The nuclear expression of four phosphorylation sites of STMN1 in LoVo and Hct116 CoCl_2_-treated cells transfected with siRNA PAK1-2133, PC, NC, and MC, respectively. **D.** Verification of the interaction between PAK1 and P-STMN1 by Co-IP in LoVo control and CoCl_2_-treated cells (a) and Hct116 control and CoCl_2_-treated cells (b). **E.** (a, b) Total protein expression of PAK1, P38MAPK, STMN1, and α/β tubulin after transfection with PTPN14 overexpression plasmid in CoCl_2_-treated LoVo and Hct116 cells. CTR, control cells; TRE, CoCl_2_-treated cells; PC, positive control; NC, negative control; MC, mock control.

The expression levels of Cdc42 interacting proteins, including cathepsin B, cathepsin D, α/β tubulin, STMN1, PTPN14, and p38MAPK, were compared before and after PAK1 knockdown. Three transfection sequences, 2133, 1228, and 629, showed that the siRNA PAK1-2133 sequence had the strongest inhibitory efficiency (Fig. 5F). No changes were observed in the expression levels of cathepsin B and cathepsin D in the control cells and PGCCs with progeny cells before and after PAK1 knockdown. The α/β tubulin level decreased after PAK1 knockdown. The expression levels of STMN1, PTPN14, and p38MAPK increased after PAK1 knockdown (Fig. 6A a, b; 6B a, b). These results confirmed that Cdc42 can regulate the expression levels of cathepsin B and cathepsin D, and PAK1 cannot influence the expression of cathepsin B and cathepsin D. Furthermore, phosphorylated STMN1 with Ser16, Ser25, Ser38, and Ser63 sites was significantly decreased after PAK1 knockdown (Fig. 6C a, b), and nuclear expression of phosphorylated STMN1 was observed at the Ser16, Ser25, Ser38, and Ser63 sites after the expression of PAK1 was decreased (Fig. 6C c, d). After knocking down PAK1, the expression level of phosphorylated STMN1 decreased and the expression level of STMN1 increased (Fig.6C). The phosphorylation of Serl6 is most significant for the acitvity of STMN1 22. In order to explore the relationship between phosphorylated STMN1 and PAK, Co-IP experiments was performed. The results of the Co-IP also confirmed the interaction between PAK1 and P-STMN1ser16 (Fig. 6D).

### PTPN14 regulates the expression of dephosphorylation of p38MAPK and regulates the expression of PAK1, α/β tubulin, and STMN1

PTPN14 expression was decreased in cells after CoCl_2_ treatment. The expression of p38MAPK and PTPN14 was upregulated after PAK1 knockdown (Fig 6A a, b; 6B a, b). To verify the relationship between PTPN14 expression and PAK1, p38MAPK, α/β tubulin, and STMN1, a PTPN14 overexpression plasmid was constructed and transfected into PGCCs with progeny cells. The western blotting results showed that the expression of PTPN14 was significantly increased (Fig. 6E a, b). After PTPN14 overexpression, the expression of PAK1, p38MAPK, α/β tubulin, and STMN1 increased, and the expression of P-STMN1 slightly decreased (Fig. 6E a, b).

### Observation of PGCCs budding to form progeny cells by laser scanning confocal microscopy

**Figure 7:**
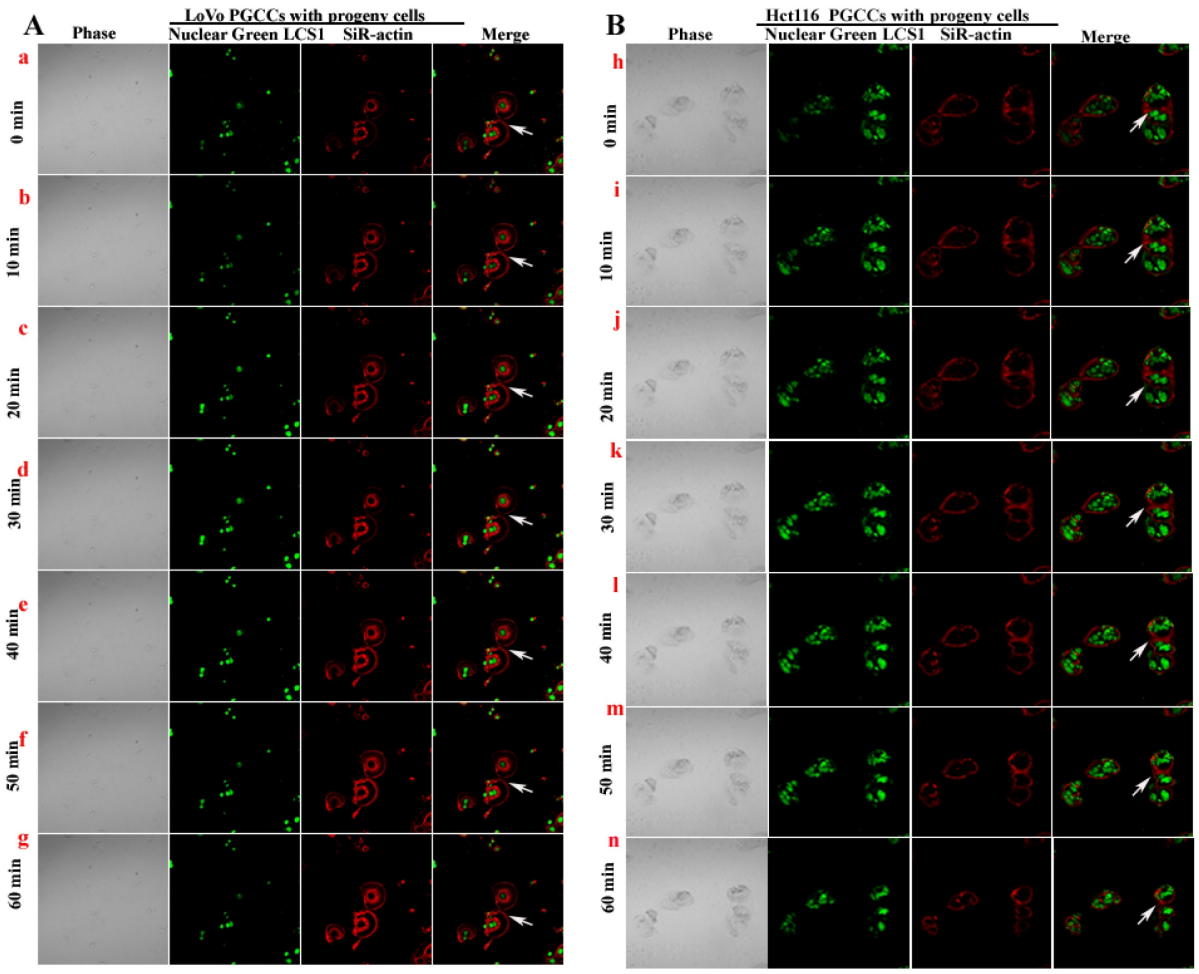
LoVo (A) and Hct116 (B) PGCCs producing progeny cells were observed and imaged using time-lapse photography under LCM.

The SiR-actin kit was used to label F-actin in living cells, and LCS1 was used to label the nucleus. The cells were observed and photographed under LCM (every 5 minutes). From the results of LCM, LoVo (Fig. 7A a-g) and Hct116 (Fig. 7B h-n) PGCCs producing progeny cells via asymmetric division were observed. The fluorescence intensity of F-actin was more intense at the position where PGCCs produced progeny cells (Fig. 7A a-n).

## Discussion

It is reported that the formation of PGCCs was asscoatied with the mitotic slippage or genomic instability intermediates 23, 24. In previous studies, we demonstrated that progeny cells generated by the asymmetric division of PGCCs had a stronger ability to invade, metastasize, and infiltrate the surrounding tissues 25, 26. In this study, we elucidated the molecular mechanisms by which Cdc42 regulates the cytoskeleton expression involved in the high invasion and metastatic ability of progeny cells derived from PGCCs.

The small GTPases Cdc42 can regulate the expression of cytoskeletal proteins which are involved in regulating cell polarity, microtubule dynamics, and asymmetric cell division 5, 27, 28. In the present study, we confirmed that Cdc42 and its related proteins in the regulation of cytoskeleton are involved in the asymmetric generation of PGCCs to generate progeny cells with enhanced invasion and migration abilities. The western blotting results showed that the expression of Cdc42 in PGCCs with progeny cells treated with CoCl_2_ was higher than that in control cells. Cell functional experiments showed that after knockdown of Cdc42, the proliferation, metastasis, and invasion abilities of LoVo and Hct116 PGCCs with progeny cells decreased. PAK1, a serine/threonine kinase, acts as a downstream target of Cdc42. Gene amplification and alterations in upstream regulators lead to aberrant PAK signaling, thereby inducing cancer development 29. Cdc42 regulating the biological behaviors of LoVo and Hct116 PGCCs with progeny cells may be associated with PAK1.

STMN1 is a microtubule-disrupting protein closely related to the cell cycle. Activated Rac/Cdc42 induces PAK1 to phosphorylate STMN1, and phosphorylated STMNs can promote the cytoskeleton rearrangement 30. The microtubule depolymerization activity of STMN1 occurs through the direct binding of two unpolymerized tubulins to form an STMN1-tubulin-STMN1 heterodimer, which increases the microtubule mutation rate through a GTP hydrolysis-dependent mechanism 31. Changes in the stathmin expression levels and phosphorylation status are important for the regulation of microtubule polymerization kinetics, particularly during cell cycle progression. STMN1 contains four phosphorylation sites (Ser16, Ser25, Ser38, and Ser63), and distinct N-terminal regions determine its subcellular localization 32. Studies have shown that PKA phosphorylates Ser16 and Ser63 in STMN1 33. STMN1 Ser25 and Ser38 have been shown to be phosphorylated by CDK1, a master regulator of M-phase progression at the G2/M transition 34, 35. The four phosphorylation sites of STMN1, namely Ser16, Ser25, Ser38, and Ser63, are involved in the regulation of various kinases during cell cycle and signal transduction cascades 36. The microtubule-depolymerizing activity of STMN1 was turned off by phosphorylation at the onset of mitosis to allow microtubule polymerization and assembly of the mitotic spindle. As cells re-enter interphase from mitosis, phosphorylated STMN1 must be reactivated by dephosphorylation 22. Several experimental studies have shown that the degree of STMN1 overexpression in tumor cells is associated with the degree of tumor malignancy 37, 38. Our study showed that the expression of STMN1 was decreased in PGCCs with progeny cells, and Cdc42/PAK1 was involved in the regulation of STMN1 phosphorylation. The western blotting results showed that after knocking down the expression of PAK1, the nuclear expression of P-STMN (Ser16, Ser25, Ser38, and Ser63) decreased. However, after the knockdown of Cdc42, P-STMN (Ser16, Ser25) was changed significantly, whereas the Ser38 and Ser63 expression levels did not change. Cdc42 directly induces the phosphorylation of Ser16 and Ser25. Phosphorylated STMN1 is located in the nucleus and promotes microtubule polymerization.

PKA1α belongs to the serine/threonine kinase superfamily and is involved in tumor transformation and growth through the CAMP/PKA signaling pathway. In normal mammalian cells, PKA1α is localized to the cytoplasm. The western blotting results showed that the expression of PKA1α was downregulated in PGCCs with progeny cells. In cancer cells, PKA1α is secreted and results in a decrease in intracellular PKA1α 39. In PGCCs with progeny cells, the downregulated expression of STMN1 led to a decrease in the activity of microtubule destruction, and an increased expression of α/β tubulin contributed to the formation of microtubule structures, which could promote the secretion of PKA1α from intracellular to extracellular space and increased sensitivity to CAMP, thereby lowering the threshold for activation of cAMP-mediated downstream effects. After reducing Cdc42 expression, the expression of PKA1α was increased. These results demonstrate that Cdc42 is involved in the direct regulation of PKA1α in PGCCs with progeny cells, mediating the processes of proliferation, metastasis, and invasion via the CAMP/PKA signaling pathway.

PTPN14, a non-receptor protein tyrosine phosphatase, is a tumor suppressor gene involved in the regulation of cell adhesion, cell proliferation, and the cytoskeleton 40. Ogata et al. demonstrated that PTPN14 negatively regulates tumor cell proliferation. However, the mechanism by which PTPN14 specifically regulates cytoskeletal proteins remains unclear 41. The western blotting and RT-PCR results showed that the expression levels of STMN1 and PTPN14 were decreased in PGCCs with progeny cells, and the downregulated expression of PTPN14 resulted in the strong migration and invasion abilities of the cells. STMN1 may act as an upstream protein of PTPN14 to regulate the expression of PTPN14 in PGCCs with progeny cells. MAPK signaling involves kinase cascades that control a wide range of cellular functions, including proliferation, stress response, and differentiation. There are four p38 isoforms: MAPK14 (p38α), MAPK11 (p38β), MAPK12 (p38γ), and MAPK13 (p38δ). p38 kinase can deliver signals through many kinases, phosphatases, transcription factors, and mRNA-binding proteins 42. PAK1 phosphorylates MAP kinase kinase 3 (MKK3) and MAP kinase kinase 3 (MKK4), which further activate p38MAPK 43. Studies have shown that p38MAPK acts as a tumor suppressor gene, negatively regulating cell cycle progression and inducing cell cycle arrest in the G2/M phase, as well as apoptosis 44. The expression of p38MAPK and PTPN14 increased after the knockdown of PAK1. The transient transfection of the PTPN14 overexpression plasmid showed that the expression of PAK1 and p38MAPK was increased in PGCCs with progeny cells. PTPN14, as a factor regulating p38MAPK activity in this pathway, plays a role in dephosphorylation and inactivation, which further induces G2/M cell cycle arrest in PGCCs with progeny cells. In addition, the western blotting results showed that the expression of p38MAPK and STMN1 increased, and the expression of P-STMN1 decreased in PGCCs with progeny cells after PTPN14 overexpression, indicating that PTPN14 could also participate in regulating the process of cytoskeleton remodeling. The dysregulation of cathepsins is an important component of tumorigenesis and tumor transformation 45. Cathepsin D activates the precursor of cathepsin B, which further activates cathepsin D and transports cathepsin B and D between cells through the actin skeleton and microtubules, thereby degrading the extracellular matrix and promoting the occurrence and development of tumors 46. In this study, we confirmed that the expression levels of cathepsin B and D are regulated by Cdc42 and are not affected by the expression of PAK1.

After the application of siRNA-Cdc42 and ML141, the expression of cathepsin B, D, and α/β tubulin was inhibited, while the expression of cathepsin B and D after siRNA-PAK1 treatment showed no significant changes, indicating that cathepsin B and D were directly regulated by Cdc42. α/β tubulin can be regulated by Cdc42 and inhibit the destruction of microtubules via activated PAK1 and the phosphorylation of STMN1. Changes in F-actin during the budding process of progeny cells produced by PGCCs were observed by LCM. The mutual activation of PAK1 and Cdc42 contributes to the directed migration of F-actin 20. According to time-lapse photography, the activity of F-actin became stronger over time, especially at the budding site of progeny cells. The results showed that the enhanced activity of F-actin was the premise of the budding process of PGCCs, and F-actin accumulation controlled the direction of cell movement at the budding site of the progeny cells.

**Figure 8:**
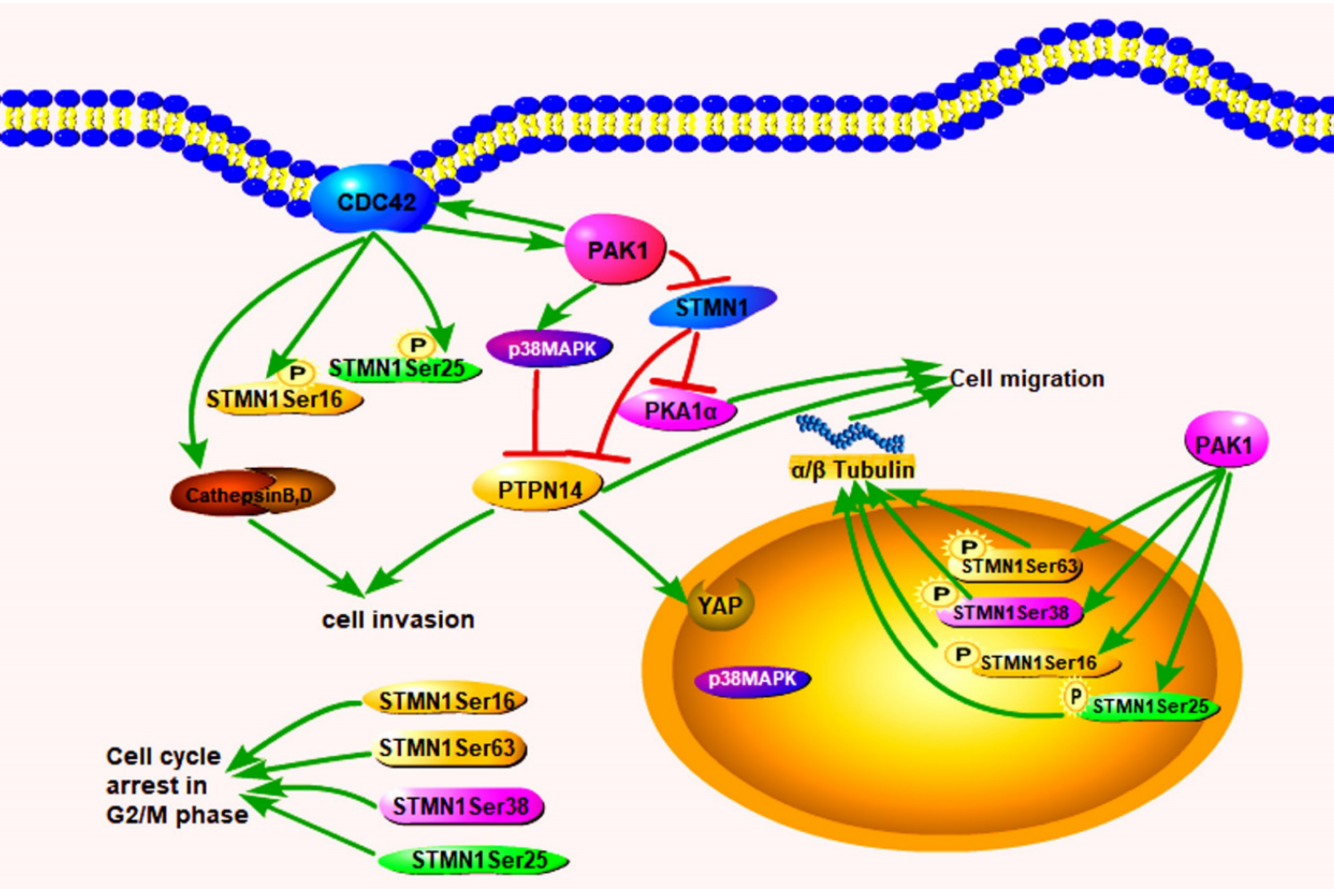
The expression of Cdc42 and PAK1 promotes the expression of p38MAPK and the phosphorylation level of STMN1, as well as the invasion and migration of CoCl_2_-treated cells. Cdc42 directly regulates its interacting proteins cathepsin B and D to participate in the invasion and migration of CoCl_2_-treated cells. Cdc42 can directly activate PAK1 and active PAK1 can phosphorylate STMN1 at sites of Ser16, Ser25, Ser38, and Ser63. Phosphorylated STMN1 can be localized in the nucleus, which further regulates the process of cytoskeleton remodeling, involved in asymmetric division of CoCl_2_-treated cells and G2/M cell cycle arrest.

In conclusion, we confirmed that increased Cdc42 and PAK1 expression can decrease the expression of STMN1 and increase the expression of phosphorylated stathmin, which is located in the nucleus of PGCCs and progeny cells to regulate cytoskeletal remodeling. Cdc42 can also bind to GTPases to activate PAK1 and induce the phosphorylation of STMN1, and the phosphorylation of the corresponding site of STMN1 leads to a decrease in microtubule destruction. PAK1 and Cdc42 contributes to the directed migration of F-actin. PAK1 can activate p38MAPK and PTPN14 dephosphorylates p38MAPK. PTPN14 could participate in the process of cytoskeleton remodeling by regulating PAK1, p38MAPK and STMN1. Cathepsin B and D, which are regulated by Cdc42, are secreted into the extracellular space through the microtubule structure to promote the migration and invasion of PGCCs with progeny cells. The pathway by which Cdc42 regulates the invasion, proliferation, and migration of PGCCs and progeny cells is shown in figure 8.

## Supplementary Material

Supplementary figures and tables.

## Abbreviations

PGCCs: polyploid tumor giant cells
EMT: epithelial-mesenchymal transition
Cdc42: cell division control protein 42
PAKs: p21-activated kinases
IARC: International Agency for Research on Cancer
VM: vasculogenic mimicry
RAC1: Ras-related protein 1
ATCC: American Type Culture Collection
WB: western blot analysis
ICC: Immunocytochemical
Co-IP: Co-immunoprecipitation
PTPN14: tyrosine-protein phosphatase non-receptor type 14
IF: immunofluorescence
PBS: phosphate buffer saline
RT-PCR: real-time quantitative PCR
STMN1: stathmin 1
PKA1α: cAMP-dependent protein kinase type 1
p38MAPK: mitogen-activated protein kinase
