# Supplementary material for: Cdc42 Regulates the Expression of Cytoskeleton and Microtubule Network Proteins to Promote Invasion and Metastasis of Progeny Cells Derived from CoCl2-induced Polyploid Giant Cancer Cells
Source: J Cancer. 2023 Jun 26;14(10):1920–34. doi: 10.7150/jca.85032 (PMC10355212; doi:10.7150/jca.85032)
Supplement: Supplementary file 1 — Supplementary figures and tables. [file jcav14p1920s1.pdf]

Supplementary figure legends

Supplementary figure 1. A. Flow cytometry analysis for cell cycle in LoVo and Hct116 cells before and after $CoCl_2$ treament. (a,b) The cell cycle analysis at G1, S, and G2 stages in LoVo control and $CoCl_2$-treated cells. (c,d) The analysis results of Hct116 control and $CoCl_2$-treated cells at G1, S, and G2 stages. B. ICC staining in LoVo and Hct116 control and $CoCl_2$-treated cells (ICC, ×200). (a–d) Cdc42 staining. (e-h) ICC staining of PAK1. (i-l) ICC staining of STMN1. (m-p) ICC staining of P-STMN-Ser 16. (q-t) ICC staining of PTPN14. C. ICC staining in LoVo and Hct116 control cells and $CoCl_2$-treated cells (200×) (a-d) α/β tubulin, (e-h) cathepsin B, (i-l) PKA1α, and (m-p) cathepsin D.

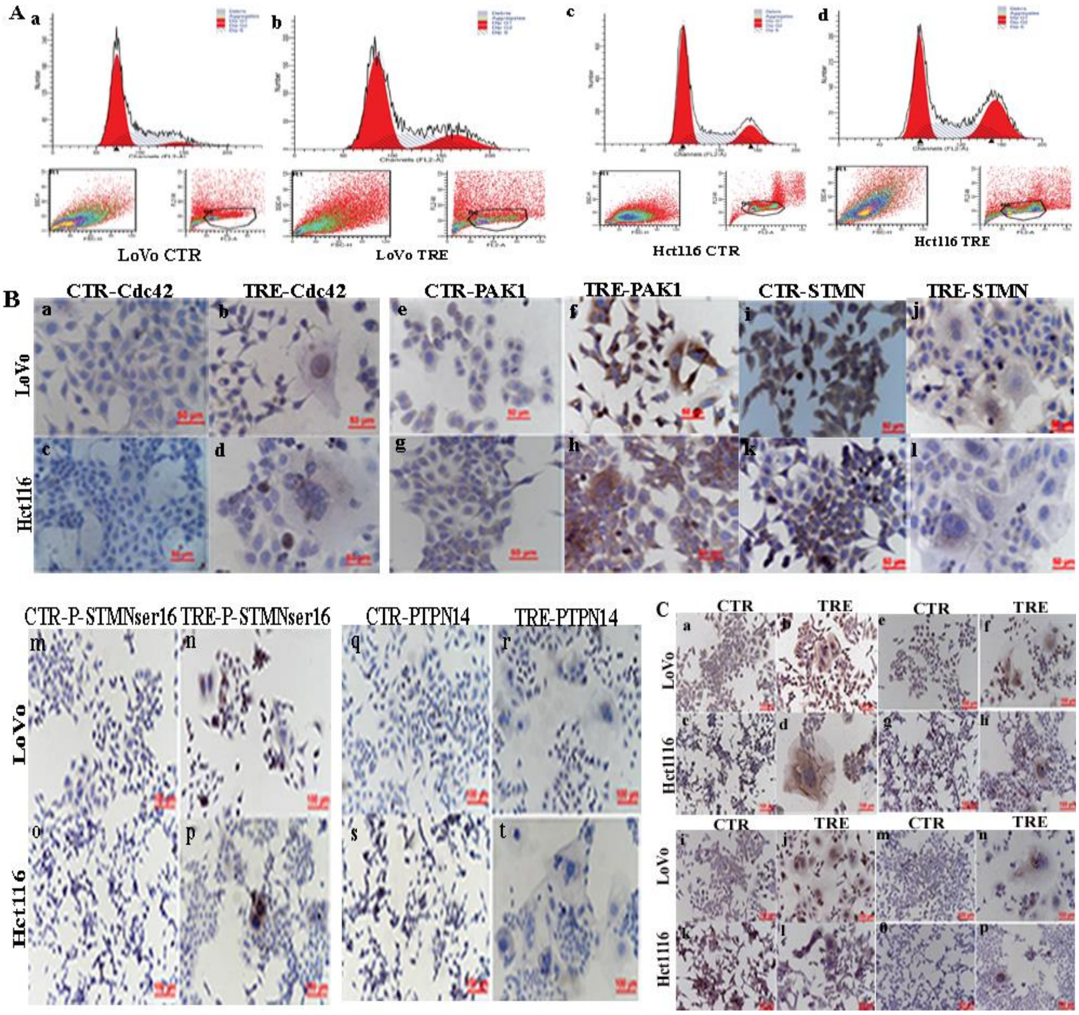

Supplementary figure 2. A. (a-h) Statistical analysis histograms of total protein expression of Cdc42, PAK1, STMN1, P-STMN1 (Ser16, Ser25, Ser38, and Ser63), and PTPN14 in LoVo and Hct116 control cells and $CoCl_2$-treated cells. **B.** Results of Co-IP in LoVo and Hct116 control cells and $CoCl_2$-treated cells**. C.**The mRNA levels of PKA1α were examined by RT-PCR in LoVo and Hct116 control and $CoCl_2$ treated cells. **D.** Statistical analysis histogram of total protein expression of Cdc42, PAK1, α/β tubulin, PKA1α, cathepsin B, cathepsin D, PTPN14, and STMN1 in LoVo and Hct116 control and PGCCs with daughter cells before and after ML141 treatment.

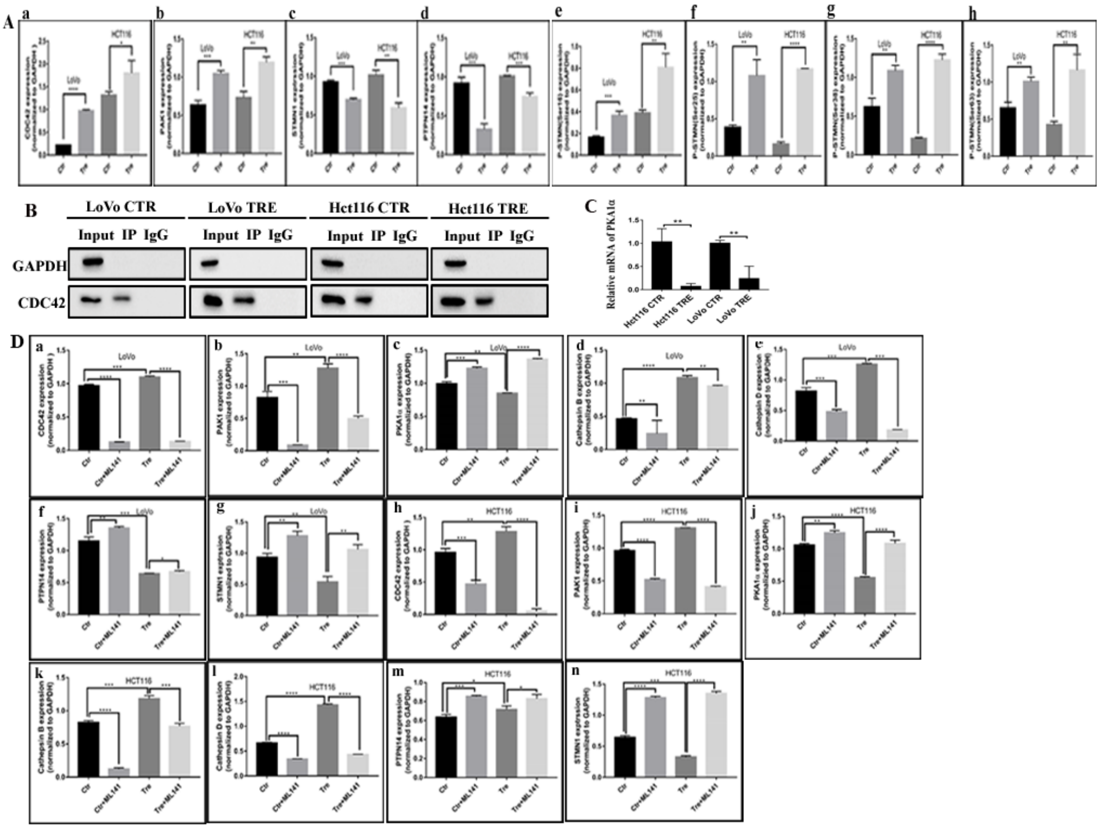

Supplementary table 1. List of siRNA used in this paper.

**Name**	**Sense (5′-3′)**	**Antisense**
Cdc42-532	CCUCUACUAUUGAGAAACUTT	AGUUUCUCAAUAGUAGAGGTT
Cdc42-369	CCGCUGAGUUAUCCACAAATT	UUUGUGGAUAACUCAGCGGTT
Cdc42-627	GUGGAGUGUUCUGCACUUATT	UAAGUGCAGAACACUCCACTT
PAK1-2133	GCAUCAAUUCCUGAAGAUUTT	AAUCUUCAGGAAUUGAUGCTT
PAK1-1228	GAUGCUUUGACCCGGAAUATT	UAUUCCGGGUCAAAGCAUCTT
PAK1-629	CCCUAAACCAUGGUUCUAATT	UUAGAACCAUGGUUUAGGGTT
β-actin	UGAAGAUCAAGAUCAUUGCdTdT	GCAAUGAUCUUGAUCUUCAdTdT
NC	UUCUCCGAACGUGUCACGUTT	ACGUGACACGUUCGGAGAATT

Supplementary table 2. List of primers used in this paper.

**Gene**	**Primer**	**Sequence (5′-3′)**
PTPN14	forward	ATGCCTTTTGGTCTGAAGCTC
	reverse	CCCTGTGCTTTCCACCGAC
α tubulin	forward	TGCTCATGGAACGTCTCTCAG
	reverse	GGCTAATAAGGCGGTTAAGGTT
β tubulin	forward	GGAGATCGTGCACATCCAG
	reverse	TCGATGCCATGCTCATCAC
GAPDH	forward	GGGAAACTGTGGCGTGAT
	reverse	GAGTGGGTGTCGCTGTTGA

Supplementary table 3. Cdc42-interacting proteins based on MS analysis of co-precipitation substrates in LoVo PGCCs with daughter cells.

Protein names	Sequence coverage [%]	Q-value	Score
**LoVo PGCC-Cdc42**			
Tubulin beta-2B chain	36	0	62.18
Keratin, type II cytoskeletal 6B	54	0	260.96
Ras-related protein Rab-10	15	0	6.2
Receptor of-activated protein C kinase 1 (Fragment)	20	0	3.66
Alpha-crystallin A2 chain	22	0	3.22
Keratin, type II cytoskeletal 1	68	0	721.75
Actin, cytoplasmic 1	79	0	887.73
Myosin-9	66	0	2013.78
Keratin, type I cytoskeletal 9	73	0	418.75
Erlin-2	56	0	290.51
Prelamin-A/C	55	0	132.18
Filamin-A	43	0	458.22
Stomatin-like protein 2, mitochondrial	51	0	127.53
Stress-70 protein, mitochondrial	41	0	118.79
Annexin A2	56	0	67.63
Ezrin	28	0	43.34
ADP/ATP translocase 2	32	0	26.46

Drebrin	13	0	28.06
Serine/threonine-protein phosphatase PGAM5	31	0	19.3
F-actin-capping protein subunit alpha-1	24	0	12.31
Actin, alpha skeletal muscle	33	0	515.39
Cystatin-A	62	0	6.37
Protein S100-A9	36	0	14.16
Cofilin-1	19	0	15.82
F-actin-capping protein subunit beta	25	0	12.83
Protein S100-A7	26	0	13.67
Poly(rC)-binding protein 2	18	0	9.37
Twinfilin-1	13	0	8.89
Galectin-7	24	0	4.6
Peroxiredoxin-2	23	0	6.5
Emerin	21	0	2.06
Prelamin-A/C	60	0	110.27
Hornerin	5	0	13.06
Trypsin-3	4	0	3.04
Pyruvate kinase PKM	29	0	28.21
Calmodulin-like protein 5	58	0	15.88
Drebrin	13	0	28.06
Liprin-alpha-3	18	0	34.68
Tropomyosin alpha-3 chain	43	0	15.91
Caveolae-associated protein 1	13	0	12.31
F-actin-capping protein subunit beta	25	0	12.83

Supplementary table 4. Cdc42-interacting proteins based on MS analysis of co-precipitation substrates in HCT116 PGCCs with daughter cells.

Protein names	Sequence coverage[%]	Q-value	Score
**HCT116 PGCC**-Cdc42			
HUMAN Unconventional myosin-Ic (Fragment)	22	0	12.15
HUMAN Heat shock cognate 71 kDa protein	40	0	168.13
HUMAN Heat shock protein beta-1	38	0	37.91
HUMAN Albumin	19	0	37.13
HUMAN Calmodulin-like protein 5	47	0	18.7
HUMAN Arginine--tRNA ligase	22	0	20.78
HUMAN Beta-enolase	8	0	13.96
HUMAN Keratin, type II cytoskeletal 73	9	0	33.21

HUMAN F-actin-capping protein subunit alpha-1	12	0	2.06
HUMAN Cytoskeleton-associated protein 4	12	0	12.37
HUMAN Actin, alpha skeletal muscle	33	0	119.06
HUMAN Tubulin beta-4B chain	58	0	394.12
HUMAN Keratin, type I cytoskeletal 9	62	0	322.99
HUMAN Ezrin	52	0	156.34
HUMAN Annexin A2	71	0	106.65
HUMAN Eukaryotic translation initiation factor 2 subunit 3	50	0	76.28
HUMAN Protein RCC2	28	0	37.22
HUMAN Protein S100-A7	24	0	18.37
HUMAN Catenin beta-1	22	0	33.65
HUMAN 14-3-3 protein theta (Fragment)	20	0	7.61
HUMAN Cathepsin D	9	0	8.64
HUMAN Serpin B6	11	0	4.33
HUMAN GTP-binding nuclear protein Ran	15	0	9.43
HUMAN Annexin A3	24	0	11.16
HUMAN Cysteine and glycine-rich protein 1	16	0	4.32
HUMAN Protein disulfide-isomerase A3 (Fragment)	32		8.96
HUMAN Ras-related protein Rab-15 (Fragment)	8	0	2.24
HUMAN Myosin-14	4	0	21.51
HUMAN Elongation factor 1-alpha 2	23	0	109.13
HUMAN Stathmin	9	0	2.5
HUMAN Stomatin-like protein 2, mitochondrial	54	0	513.26
HUMAN Erlin-2	53	0	282.37

HUMAN Cofilin-1	32	0	18.6
HUMAN Septin-2 (Fragment)	38	0	4.09
HUMAN Cathepsin B	12	0	4.02
HUMAN ADP/ATP translocase 2	48	0	70.75
HUMAN Liprin-alpha-3	30	0	105.26
HUMAN Eukaryotic translation initiation factor 2 subunit 3	50	0	76.28
HUMAN Peroxiredoxin-1	52	0	43.02
HUMAN Annexin A1	51	0	60.8
HUMAN Actin-depolymerizing factor	44	0	26.47
HUMAN Microtubule-associated protein 4	10	0	26.33
HUMAN Protein RCC2	28	0	37.22
HUMAN Nucleoside diphosphate kinase A	31	0	23.63
HUMAN Serpin B3	21	0	22.9
HUMAN Fatty acid-binding protein 5	39	0	13.4
HUMAN Caspase-14	16	0	9.75
